# *N*-(4-Chloro­pyridin-2-yl)-*N*-meth­oxy­methyl-4-methyl­benzene­sulfonamide

**DOI:** 10.1107/S1600536810048336

**Published:** 2010-11-27

**Authors:** Stefanie Bühler, Dieter Schollmeyer, Wolfgang Albrecht, Stefan Laufer

**Affiliations:** a Eberhard-Karls-University Tübingen, Auf der Morgenstelle 8, 72076 Tübingen, Germany; b University Mainz, Institute of Organic Chemistry, Duesbergweg 10-14, 55099 Mainz, Germany; c c-a-i-r biosciences GmbH, Paul-Ehrlich-Strasse 15, 72076 Tübingen, Germany

## Abstract

In the crystal structure of the title compound, C_14_H_15_ClN_2_O_3_S, each mol­ecule is connected *via* inter­molecular C—H⋯O hydrogen bonds to three further mol­ecules, generating a three-dimensional network. The 4-methyl­phenyl­sulfonyl ring forms a dihedral angle of 40.7 (2)° with the 4-chloro­pyridine ring.

## Related literature

For the biological activity of 2-alkyl­amino­pyridinyl or 2-acyl­amino­pyridinyl imidazole derivatives as p38α MAPK inhibitors, see: Laufer *et al.* (2008 ▶, 2010 ▶); Ziegler *et al.* (2009 ▶). For general background to protecting groups, see: Kocieński (2005 ▶). For the preparation of the *N*-protected 4-chloro­pyridine, see: Berliner & Belecki (2005 ▶); Sciotti *et al.* (2005 ▶); Shi & Wang (2002 ▶).
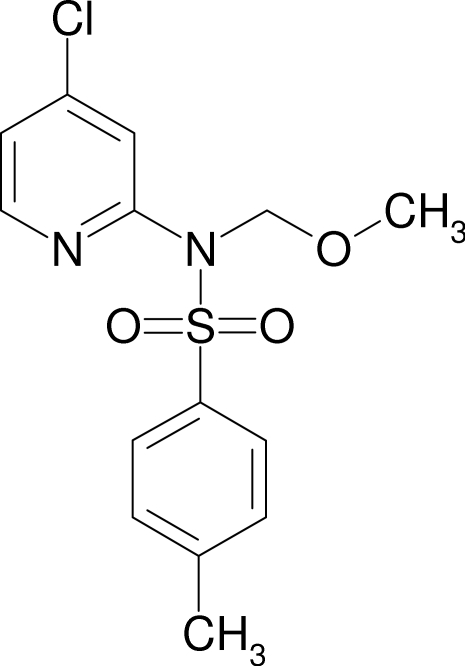


## Experimental

### 

#### Crystal data

C_14_H_15_ClN_2_O_3_S
*M* *_r_* = 326.79
Orthorhombic, 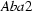

*a* = 15.1651 (10) Å
*b* = 22.7953 (13) Å
*c* = 8.9132 (6) Å
*V* = 3081.2 (3) Å^3^
*Z* = 8
Cu *K*α radiation
μ = 3.57 mm^−1^
*T* = 193 K
0.30 × 0.30 × 0.20 mm

#### Data collection

Enraf–Nonius CAD-4 diffractometer
Absorption correction: ψ scan (*CORINC*; Dräger & Gattow, 1971 ▶) *T*_min_ = 0.736, *T*_max_ = 0.999
2950 measured reflections
2742 independent reflections
2659 reflections with *I* > 2σ(*I*)
*R*_int_ = 0.080
3 standard reflections every 60 min  intensity decay: 2%

#### Refinement

*R*[*F* ^2^ > 2σ(*F* ^2^)] = 0.053
*wR*(*F* ^2^) = 0.158
*S* = 1.11
2742 reflections
192 parameters
1 restraint
H-atom parameters constrained
Δρ_max_ = 0.66 e Å^−3^
Δρ_min_ = −0.53 e Å^−3^
Absolute structure: Flack (1983 ▶), 1176 Friedel pairs
Flack parameter: 0.02 (3)

### 

Data collection: *CAD-4 Software* (Enraf–Nonius, 1989 ▶); cell refinement: *CAD-4 Software*; data reduction: *CORINC* (Dräger & Gattow, 1971 ▶); program(s) used to solve structure: *SIR97* (Altomare *et al.*, 1999 ▶); program(s) used to refine structure: *SHELXL97* (Sheldrick, 2008 ▶); molecular graphics: *PLATON* (Spek, 2009 ▶); software used to prepare material for publication: *PLATON*.

## Supplementary Material

Crystal structure: contains datablocks I, global. DOI: 10.1107/S1600536810048336/bt5411sup1.cif

Structure factors: contains datablocks I. DOI: 10.1107/S1600536810048336/bt5411Isup2.hkl

Additional supplementary materials:  crystallographic information; 3D view; checkCIF report

## Figures and Tables

**Table 1:** Hydrogen-bond geometry (Å, °)

*D*—H⋯*A*	*D*—H	H⋯*A*	*D*⋯*A*	*D*—H⋯*A*
C7—H7*C*⋯O9^i^	0.98	2.59	3.512 (6)	157
C16—H16⋯O13^ii^	0.95	2.56	3.485 (4)	165
C18—H18⋯O10^iii^	0.95	2.50	3.098 (5)	121
C19—H19⋯O10^iii^	0.95	2.48	3.112 (5)	124

Symmetry codes: (i) 
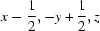
; (ii) 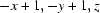; (iii) 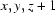.

**Fig. 1.**
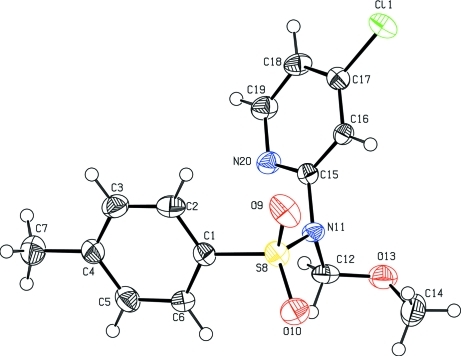
View of compound I. Displacement ellipsoids are drawn at the 50% probability level.

## supplementary crystallographic information

### Comment

In recent years, compounds with the 2-aminopyridine moiety exhibited interesting biological activities like the 2-alkylaminopyridinyl or 2-acylaminopyridinyl imidazole derivatives as p38α mitogen-activated protein kinase (p38α MAPK) inhibitors. The N-protected 4-chloropyridine is an important precursor to block the nucleophilic and basic properties of the amino-group in the C2 position of the pyridine ring. The analysis of the crystal structure shows that the both aromatic C19—H– and C18—H-groups of the 4-chloropyridine ring of one molecule interact with the oxygen-atom O10 of the sulfonyl group of another molecule by the building of a bidentate intermolecular hydrogen bond C—H···O, whereas the O10···H19 distance is 2.48 Å. The length of the second hydrogen bond O10···H18 is 2.50 Å. Furthermore, the aromatic C16—H group of the 4-chloropyridine ring forms an intermolecular C16—H16···O13 hydrogen bond (2.56 Å) to the oxygen atom O13 of the methoxymethyl moiety of a third molecule. An additional hydrogen bond was observed between the methyl-group C7—H_3_ of the 4-methylphenylsulfonylring and the oxygen-atom O9 of the sulfonyl group of a further molecule, whereas the O9_a···H7C distance is 2.59 Å. The 4-methylphenylsulfonyl ring forms a dihedral angle of 40.7 (2)° to the 4-chloropyridine ring.

### Experimental

Synthesis of chloromethyl methyl ether as a solution of toluene: To a solution of dimethoxymethane (44.3 ml, 0.50 mol, 1 equiv) and Zn(OAc)_2_ (9.2 mg, 0.01%) in toluene (133 ml) was added acetyl chloride (35.5 ml, 0.50 mol, 1 equiv). During the next 15 min, the reaction mixture warmed slowly at T = 318 K, and then cooled to ambient temperature over 3 h. The progress was again monitored until NMR analysis indicated complete conversion. The solution of MOMCl in toluene prepared using this stoichiometry is approximately 2.1 *M*.

Synthesis of *N*-(4-chloropyridin-2-yl)-4-methylbenzenesulfonamide: 2-amino- 4-chloropyridine (20.1 g, 156 mmol. 1 equiv) and 4-toluenesulfonyl chloride (32.4 g, 168 mmol, 1.1 equiv) were dissolved in dry pyridine (70 ml) and heated at T = 353 K for 5 h. After cooling to room temperature, water was added and the compound *N*-(4-chloropyridin-2-yl)-4-methylbenzenesulfonamide dropped down as a beige solid with high analytical quality, which was filtered off and washed with water (30.6 g, 70.8%).

Synthesis of *N*-(4-chloropyridin-2-yl)- *N*-(methoxymethyl)- 4-methylbenzenesulfon- amide: Under a nitrogen atmosphere, *N*-(4-chloropyridin-2-yl)- 4-methylbenzene- sulfonamide (20.0 g, 71 mmol, 1 equiv) was added to a suspension of NaH (4.2 g, 104 mmol, 1.5 equiv) in anhydrous THF (200 ml) with stirring. The resulting reaction mixture was stirred for 20 min, and then the solution of methoxymethyl chloride in toluene (52.1 ml, 1.5 equiv) was slowly added. The mixture was stirred for 3 h and then an aqueous saturated solution of NH_4_Cl was added. After separation, the aqueous layer was extracted with EtOAc, dried over Na_2_SO_4 _and evaporated. After treatment with hexane, the compound *N*-(4-chloropyridin-2-yl)- *N*-(methoxymethyl)-4- methylbenzene sulfonamide was obtained as the main product of the reaction (15.8 g, 69.7%) and dropped down as a pale yellow solid, whereas the compound *N*-(4-chloropyridin-2-yl)-*N*-tosylacetamide was isolated from the filtrate as the byproduct (15.4%). Suitable crystals of the compound *N*-(4-chloropyridin-2-yl)-N– (methoxymethyl)-4-methylbenzenesulfonamide for X-ray were obtained by slow evaporation at T = 298 K of a solution of EtOAc.

### Refinement

Hydrogen atoms   were placed at calculated positions with C—H = 0.95 Å (aromatic) or 0.98–0.99 Å (*sp*^3^ C-atom). They were refined in the riding-model approximation with isotropic displacement parameters (set at 1.2–1.5 times of the *U*_eq_ of the parent atom). The absolute structure was determined on the basis of 1176 Friedel pairs.

### Crystal data

**Table:**

C_14_H_15_ClN_2_O_3_S	*F*(000) = 1360
*M**_r_* = 326.79	*D*_x_ = 1.409 Mg m^−^^3^
Orthorhombic, *A**b**a*2	Cu *K*α radiation, λ = 1.54178 Å
Hall symbol: A 2 -2ac	Cell parameters from 25 reflections
*a* = 15.1651 (10) Å	θ = 65–69°
*b* = 22.7953 (13) Å	µ = 3.57 mm^−^^1^
*c* = 8.9132 (6) Å	*T* = 193 K
*V* = 3081.2 (3) Å^3^	Block, colourless
*Z* = 8	0.30 × 0.30 × 0.20 mm

### Data collection

**Table:**

Enraf–Nonius CAD-4 diffractometer	2659 reflections with *I* > 2σ(*I*)
Radiation source: rotating anode	*R*_int_ = 0.080
graphite	θ_max_ = 70.0°, θ_min_ = 3.9°
ω/2θ scans	*h* = −18→18
Absorption correction: ψ scan (*CORINC*; Dräger & Gattow, 1971)	*k* = −27→27
*T*_min_ = 0.736, *T*_max_ = 0.999	*l* = −10→10
2950 measured reflections	3 standard reflections every 60 min
2742 independent reflections	intensity decay: 2%

### Refinement

**Table:**

Refinement on *F*^2^	Secondary atom site location: difference Fourier map
Least-squares matrix: full	Hydrogen site location: inferred from neighbouring sites
*R*[*F*^2^ > 2σ(*F*^2^)] = 0.053	H-atom parameters constrained
*wR*(*F*^2^) = 0.158	*w* = 1/[σ^2^(*F*_o_^2^) + (0.1234*P*)^2^ + 0.8389*P*] where *P* = (*F*_o_^2^ + 2*F*_c_^2^)/3
*S* = 1.11	(Δ/σ)_max_ < 0.001
2742 reflections	Δρ_max_ = 0.66 e Å^−^^3^
192 parameters	Δρ_min_ = −0.53 e Å^−^^3^
1 restraint	Absolute structure: Flack (1983), 1176 Friedel pairs
Primary atom site location: structure-invariant direct methods	Flack parameter: 0.02 (3)

### Special details

**Table:**

Geometry. All e.s.d.'s (except the e.s.d. in the dihedral angle between two l.s. planes) are estimated using the full covariance matrix. The cell e.s.d.'s are taken into account individually in the estimation of e.s.d.'s in distances, angles and torsion angles; correlations between e.s.d.'s in cell parameters are only used when they are defined by crystal symmetry. An approximate (isotropic) treatment of cell e.s.d.'s is used for estimating e.s.d.'s involving l.s. planes.
Refinement. Refinement of *F*^2^ against ALL reflections. The weighted *R*-factor *wR* and goodness of fit *S* are based on *F*^2^, conventional *R*-factors *R* are based on *F*, with *F* set to zero for negative *F*^2^. The threshold expression of *F*^2^ > σ(*F*^2^) is used only for calculating *R*-factors(gt) *etc*. and is not relevant to the choice of reflections for refinement. *R*-factors based on *F*^2^ are statistically about twice as large as those based on *F*, and *R*- factors based on ALL data will be even larger.

### Fractional atomic coordinates and isotropic or equivalent isotropic displacement parameters (Å^2^)

**Table:**

	*x*	*y*	*z*	*U*_iso_*/*U*_eq_	
Cl1	0.64226 (5)	0.45727 (4)	0.66220 (13)	0.0400 (3)	
C1	0.3696 (2)	0.29193 (14)	0.2257 (4)	0.0283 (7)	
C2	0.4078 (2)	0.25319 (17)	0.3272 (5)	0.0367 (8)	
H2	0.4671	0.2585	0.3591	0.044*	
C3	0.3586 (3)	0.20715 (17)	0.3805 (5)	0.0386 (9)	
H3	0.3854	0.1794	0.4457	0.046*	
C4	0.2699 (2)	0.20019 (15)	0.3411 (5)	0.0350 (8)	
C5	0.2336 (2)	0.23969 (15)	0.2376 (5)	0.0379 (8)	
H5	0.1739	0.2351	0.2075	0.045*	
C6	0.2828 (2)	0.28515 (14)	0.1785 (5)	0.0336 (8)	
H6	0.2578	0.3112	0.1071	0.040*	
C7	0.2156 (3)	0.15130 (19)	0.4026 (6)	0.0501 (11)	
H7A	0.2187	0.1517	0.5124	0.075*	
H7B	0.2382	0.1138	0.3650	0.075*	
H7C	0.1542	0.1562	0.3709	0.075*	
S8	0.42918 (6)	0.35362 (4)	0.16453 (10)	0.0329 (3)	
O9	0.52102 (18)	0.34292 (13)	0.1894 (4)	0.0468 (8)	
O10	0.3980 (2)	0.37013 (14)	0.0185 (3)	0.0487 (8)	
N11	0.40200 (19)	0.40858 (12)	0.2779 (3)	0.0267 (6)	
C12	0.3183 (2)	0.43980 (16)	0.2508 (4)	0.0331 (8)	
H12A	0.2907	0.4244	0.1582	0.040*	
H12B	0.2774	0.4321	0.3352	0.040*	
O13	0.33051 (19)	0.49934 (12)	0.2365 (4)	0.0437 (7)	
C14	0.3640 (3)	0.5145 (2)	0.0901 (7)	0.0544 (12)	
H14B	0.3211	0.5028	0.0134	0.082*	
H14A	0.4198	0.4940	0.0727	0.082*	
H14C	0.3736	0.5569	0.0847	0.082*	
C15	0.4331 (2)	0.40624 (14)	0.4291 (4)	0.0246 (7)	
C16	0.5156 (2)	0.42870 (12)	0.4611 (4)	0.0243 (6)	
H16	0.5525	0.4442	0.3847	0.029*	
C17	0.5417 (2)	0.42749 (14)	0.6091 (4)	0.0290 (7)	
C18	0.4872 (3)	0.40358 (17)	0.7178 (4)	0.0344 (8)	
H18	0.5044	0.4021	0.8202	0.041*	
C19	0.4068 (2)	0.38197 (16)	0.6710 (5)	0.0350 (8)	
H19	0.3691	0.3651	0.7445	0.042*	
N20	0.3782 (2)	0.38313 (13)	0.5288 (4)	0.0316 (6)	

### Atomic displacement parameters (Å^2^)

**Table:**

	*U*^11^	*U*^22^	*U*^33^	*U*^12^	*U*^13^	*U*^23^
Cl1	0.0311 (4)	0.0470 (5)	0.0418 (5)	−0.0051 (3)	−0.0052 (4)	−0.0089 (4)
C1	0.0281 (16)	0.0298 (15)	0.0271 (17)	−0.0020 (12)	0.0027 (14)	−0.0038 (13)
C2	0.0302 (16)	0.0454 (19)	0.0343 (18)	0.0061 (14)	−0.0061 (17)	−0.0020 (16)
C3	0.043 (2)	0.0359 (18)	0.037 (2)	0.0076 (15)	−0.0078 (16)	0.0010 (16)
C4	0.0405 (19)	0.0314 (16)	0.0332 (19)	−0.0040 (14)	−0.0020 (18)	−0.0023 (14)
C5	0.0307 (18)	0.0373 (17)	0.046 (2)	−0.0041 (14)	−0.0032 (18)	−0.0027 (17)
C6	0.0341 (16)	0.0329 (15)	0.0337 (19)	−0.0008 (12)	−0.0088 (17)	0.0037 (15)
C7	0.062 (3)	0.043 (2)	0.045 (3)	−0.0116 (19)	−0.001 (2)	0.0057 (18)
S8	0.0347 (4)	0.0401 (4)	0.0238 (4)	−0.0081 (3)	0.0091 (4)	−0.0037 (4)
O9	0.0299 (12)	0.0588 (16)	0.052 (2)	−0.0067 (12)	0.0168 (12)	−0.0185 (14)
O10	0.067 (2)	0.0595 (17)	0.0194 (13)	−0.0161 (16)	0.0044 (14)	0.0030 (12)
N11	0.0291 (13)	0.0324 (13)	0.0188 (13)	−0.0033 (10)	−0.0015 (11)	0.0028 (11)
C12	0.0265 (16)	0.0472 (19)	0.0255 (17)	0.0007 (14)	−0.0053 (16)	0.0013 (15)
O13	0.0439 (15)	0.0426 (14)	0.0445 (16)	0.0048 (12)	−0.0128 (15)	−0.0027 (13)
C14	0.050 (3)	0.043 (2)	0.070 (3)	−0.0053 (17)	−0.002 (2)	0.018 (2)
C15	0.0253 (15)	0.0247 (15)	0.0239 (16)	0.0003 (11)	0.0014 (13)	−0.0018 (12)
C16	0.0252 (14)	0.0250 (14)	0.0226 (15)	−0.0002 (11)	0.0027 (13)	0.0016 (13)
C17	0.0258 (14)	0.0288 (14)	0.0326 (18)	0.0019 (13)	−0.0004 (15)	−0.0028 (13)
C18	0.0368 (19)	0.047 (2)	0.0200 (15)	0.0022 (15)	0.0022 (15)	0.0020 (15)
C19	0.0340 (17)	0.0465 (18)	0.0245 (17)	−0.0017 (14)	0.0047 (18)	0.0043 (17)
N20	0.0295 (13)	0.0381 (14)	0.0273 (16)	−0.0027 (12)	0.0009 (13)	0.0052 (13)

### Geometric parameters (Å, °)

**Table:**

Cl1—C17	1.736 (4)	N11—C15	1.429 (4)
C1—C6	1.391 (5)	N11—C12	1.475 (4)
C1—C2	1.391 (5)	C12—O13	1.376 (5)
C1—S8	1.758 (3)	C12—H12A	0.9900
C2—C3	1.372 (6)	C12—H12B	0.9900
C2—H2	0.9500	O13—C14	1.442 (6)
C3—C4	1.400 (5)	C14—H14B	0.9800
C3—H3	0.9500	C14—H14A	0.9800
C4—C5	1.402 (6)	C14—H14C	0.9800
C4—C7	1.490 (5)	C15—N20	1.327 (5)
C5—C6	1.381 (5)	C15—C16	1.381 (4)
C5—H5	0.9500	C16—C17	1.378 (5)
C6—H6	0.9500	C16—H16	0.9500
C7—H7A	0.9800	C17—C18	1.385 (5)
C7—H7B	0.9800	C18—C19	1.380 (5)
C7—H7C	0.9800	C18—H18	0.9500
S8—O9	1.431 (3)	C19—N20	1.340 (5)
S8—O10	1.435 (3)	C19—H19	0.9500
S8—N11	1.662 (3)		
			
C6—C1—C2	121.4 (3)	C15—N11—S8	117.6 (2)
C6—C1—S8	118.8 (3)	C12—N11—S8	118.5 (2)
C2—C1—S8	119.7 (3)	O13—C12—N11	112.0 (3)
C3—C2—C1	119.0 (3)	O13—C12—H12A	109.2
C3—C2—H2	120.5	N11—C12—H12A	109.2
C1—C2—H2	120.5	O13—C12—H12B	109.2
C2—C3—C4	121.5 (4)	N11—C12—H12B	109.2
C2—C3—H3	119.3	H12A—C12—H12B	107.9
C4—C3—H3	119.3	C12—O13—C14	111.6 (3)
C3—C4—C5	118.1 (3)	O13—C14—H14B	109.5
C3—C4—C7	121.6 (4)	O13—C14—H14A	109.5
C5—C4—C7	120.3 (4)	H14B—C14—H14A	109.5
C6—C5—C4	121.4 (3)	O13—C14—H14C	109.5
C6—C5—H5	119.3	H14B—C14—H14C	109.5
C4—C5—H5	119.3	H14A—C14—H14C	109.5
C5—C6—C1	118.7 (3)	N20—C15—C16	125.3 (3)
C5—C6—H6	120.7	N20—C15—N11	116.1 (3)
C1—C6—H6	120.7	C16—C15—N11	118.7 (3)
C4—C7—H7A	109.5	C17—C16—C15	116.8 (3)
C4—C7—H7B	109.5	C17—C16—H16	121.6
H7A—C7—H7B	109.5	C15—C16—H16	121.6
C4—C7—H7C	109.5	C16—C17—C18	120.4 (3)
H7A—C7—H7C	109.5	C16—C17—Cl1	120.4 (3)
H7B—C7—H7C	109.5	C18—C17—Cl1	119.2 (3)
O9—S8—O10	120.4 (2)	C19—C18—C17	117.1 (3)
O9—S8—N11	106.00 (16)	C19—C18—H18	121.4
O10—S8—N11	105.79 (18)	C17—C18—H18	121.4
O9—S8—C1	108.41 (18)	N20—C19—C18	124.4 (3)
O10—S8—C1	108.76 (18)	N20—C19—H19	117.8
N11—S8—C1	106.68 (15)	C18—C19—H19	117.8
C15—N11—C12	117.2 (3)	C15—N20—C19	116.0 (3)
			
C6—C1—C2—C3	0.5 (6)	O10—S8—N11—C12	35.5 (3)
S8—C1—C2—C3	176.2 (3)	C1—S8—N11—C12	−80.2 (3)
C1—C2—C3—C4	−3.4 (6)	C15—N11—C12—O13	83.7 (4)
C2—C3—C4—C5	3.9 (6)	S8—N11—C12—O13	−125.7 (3)
C2—C3—C4—C7	−178.1 (4)	N11—C12—O13—C14	78.1 (4)
C3—C4—C5—C6	−1.6 (6)	C12—N11—C15—N20	56.6 (4)
C7—C4—C5—C6	−179.7 (4)	S8—N11—C15—N20	−94.3 (3)
C4—C5—C6—C1	−1.1 (6)	C12—N11—C15—C16	−122.1 (3)
C2—C1—C6—C5	1.7 (6)	S8—N11—C15—C16	86.9 (3)
S8—C1—C6—C5	−174.1 (3)	N20—C15—C16—C17	−0.9 (5)
C6—C1—S8—O9	−163.5 (3)	N11—C15—C16—C17	177.7 (3)
C2—C1—S8—O9	20.7 (4)	C15—C16—C17—C18	1.2 (5)
C6—C1—S8—O10	−30.9 (3)	C15—C16—C17—Cl1	−177.5 (2)
C2—C1—S8—O10	153.3 (3)	C16—C17—C18—C19	−0.5 (5)
C6—C1—S8—N11	82.8 (3)	Cl1—C17—C18—C19	178.2 (3)
C2—C1—S8—N11	−93.0 (3)	C17—C18—C19—N20	−0.6 (6)
O9—S8—N11—C15	−45.1 (3)	C16—C15—N20—C19	−0.1 (5)
O10—S8—N11—C15	−174.0 (3)	N11—C15—N20—C19	−178.8 (3)
C1—S8—N11—C15	70.3 (3)	C18—C19—N20—C15	0.9 (6)
O9—S8—N11—C12	164.4 (3)		

### Hydrogen-bond geometry (Å, °)

**Table:**

*D*—H···*A*	*D*—H	H···*A*	*D*···*A*	*D*—H···*A*
C7—H7C···O9^i^	0.98	2.59	3.512 (6)	157
C16—H16···O13^ii^	0.95	2.56	3.485 (4)	165
C18—H18···O10^iii^	0.95	2.50	3.098 (5)	121
C19—H19···O10^iii^	0.95	2.48	3.112 (5)	124

Symmetry codes: (i) *x*−1/2, −*y*+1/2, *z*; (ii) −*x*+1, −*y*+1, *z*; (iii) *x*, *y*, *z*+1.
